# What place ‘capacity’ in the criminal law relating to sex post *JB*?

**DOI:** 10.1016/j.ijlp.2022.101843

**Published:** 2022-11-12

**Authors:** Alex Ruck Keene Kc Hon, Allegra Enefer

**Affiliations:** 1 Barrister, 39 Essex Chambers, London, UK; Visiting Professor, King’s College London, Dickson Poon School of Law, UK; 2 Student at City, University of London, UK; Research Assistant at the London School of Economics and Political Science, UK

**Keywords:** Sexual capacity, Mental capacity law, Sexual Offences Act 2003, Mental Capacity Act 2005, Sexual consent

## Abstract

The term ‘capacity’ has come to assume a variety of meanings in the law of England and Wales, and the failure of statutes and judges to specify its meaning and application across the civil and criminal law leads to problems. Nowhere is this perhaps clearer than in the law relating to sexual capacity. This paper begins with an overview of two streams of law on sexual capacity in the civil and criminal law. The first stream traces through the criminal law provisions of the Sexual Offences Act 2003, the work by the Law Commission which led to its enactment, and the ways in which its provisions have been applied by the courts in practice; and the second examines the Mental Capacity Act 2005 and its parallel application by the civil courts. We illustrate how the case of *A Local Authority v JB* [2021] UKSC 52 brought these problems to the fore, as the Supreme Court was at last confronted with the differences between the definition and use of the term ‘capacity’ by the civil and criminal law on sexual capacity. We suggest that the decision made by the Supreme Court in JB has left open terrain which ought to be used to reframe, or perhaps even replace, the concept of ‘capacity’ within the criminal law on sexual capacity.^1^

## 1 Introduction

‘“When I use a word... it means just what I choose it to mean” Humpty Dumpty said’ (Lewis Carroll, *Through the Looking Glass and What Alice Found There*, 1872).

“Capacity” is, in many ways, an Alice in Wonderland word. It has (at least) three different meanings within the law. The first is legal capacity, i.e., classification as an actor before the law.^2^ The second is mental capacity, i.e., a person’s functional – cognitive – abilities to decide and act; the sense in which it is used within the Mental Capacity Act 2005. The third is a broader sense of ability, not necessarily tied to cognitive abilities, for example in the consideration of a person’s parenting ability for purposes of child-care proceedings (Ward, Brown, Hyde-Dryden, 2014).

Perhaps illustrating that (as so often) Lewis Carroll was making a serious point, the failure of statutes and judges to follow Humpty Dumpty’s approach in choosing what they want the word ‘capacity’ to mean leads to problems. Nowhere is this perhaps clearer than in the context of sexual capacity in England & Wales. Two (apparently) overlapping statutes and streams of case law address different aspects of the question of whether valid consent to sexual activity can be or has been given. Both use the word ‘capacity.’ And neither is using it to mean the same thing. In this article, we trace how the criminal law and the civil law have conceptualised the concept of sexual capacity, and how the statutory formulation of those concepts have fared before the courts. Looking back at how these conceptions were developed and applied, and the different policy concerns that have been in play, gives us an opportunity to clarify the conceptual positions, to tease out whether the policy concerns are justified, and also to ask whether and how the law could move forward. We then turn to an analysis of the Supreme Court decision in the case of *A Local Authority v JB* [2021] UKSC 52 (‘*JB*’), in which the problem of the misaligned development of the criminal and civil law was brought to the fore. We suggest that the Supreme Court has provided the opportunity, in effect, to start again, and we set out some modest proposals for how we might do so. We focus on the criminal field, not least because we suggest that the problem is both more acute and less recognised.^3^

We make two preliminary points. The first is as to the scope of our discussion, which is primarily addressed to the position involving sexual activity between two people, one, or both, of whom may have impaired decision-making capacity. A person with impaired decision-making capacity could be either the initiator of such activity or (for want of a better word), the recipient of such activity; in reality, it might well be that for many purposes, most of the time, it is irrelevant which. One of the problems (it might be thought) with how the law has evolved, in both the civil, but in particular the criminal, sphere, is that it starts to be necessary to look closely at what role the person is playing, and through the rear view mirror of the criminal law to decide which one is the perpetrator and which is the victim. Such an approach, and such language, may be necessary, but it does not mean either that the language always feels right, or (perhaps more pertinently) that the underlying position feels right.

The second is that, whilst our focus is on England & Wales, we suggest that the issues that we discuss are of broader relevance. This is particularly so given the intense focus on the concepts of legal and mental capacity in consequence of the requirement contained in Article 12(2) of the UN Convention on the Rights of Persons with Disabilities (‘UNCRPD’) for States to recognise the right of disabled people to enjoy legal capacity on an equal basis with others.^4^

## 2 The road to the Sexual Offences Act 2003

Questions of whether and how the law should treat the legal ability of those with cognitive impairments to give consent to sexual activity troubled the criminal courts erratically from the middle of the 19th century. The meaning of “consent” in the sexual context was not, as a general rule, the focus of statute; rather it was left to the common law. As vividly described by Ralph Sandland (2013), the approach of the criminal courts to the question of consent in the context of cognitive impairments was characterised by attitudes which are deeply distasteful when judged by modern day standards.^5^ By the early 20^th^ century, the legislature had become involved, deeming an entire class of individuals – those (in effect) with severe learning disabilities - simply legally incapable of giving consent to any sexual activity, such that any sexual activity with such a person was an offence.^6^

For present purposes, we pick up the detailed analysis of the story in 1991, when the Law Commission turned its attention to how the law should respond to cognitive impairments in the context of sexual decision-making. In 1991, the Law Commission published a preliminary consultation paper (Law Commission, 1991). It noted that, as with marriage, sexual intercourse:

*must be a matter for personal choice by the individual, so that if he or she is incapable of making the decision, no-one may make it for them. The common law test of capacity to consent to sexual intercourse in general follows the usual form, that the person concerned must be capable of understanding what is proposed and its implications and exercising choice*.

(Law Commission, 1991, para 2.27). [^7^]

The Law Commission noted that “*Statutory limitations have* […]*, been imposed upon the capacity of certain groups of people to give a valid consent to sexual intercourse with the aim of protecting people with mental disorder from exploitation and abuse.”* As it identified:

*One problem with these provisions is that they may cover people who are in fact capable of giving a real consent to intercourse or other sexual activity, but have a statutory incapacity imposed upon them by the criminal law. The men involved in these cases may often be handicapped themselves, and it seems unfair that they should automatically be at risk of prosecution if there has been no exploitation involved. In some circumstances, these provisions of the criminal law could be seen as imposing an unwarranted fetter upon the freedom of mentally incapacitated people.^8^ They can also pose problems for staff who may fear, even if they do not risk, prosecution for aiding and abetting.*

(Law Commission, 1991, para 2.27).

At this stage, the Law Commission simply outlined the problem and did not outline what it considered the solution to be. Nor was such a solution forthcoming in its move towards its final report, *Mental Incapacity: Item 9 of the fourth programme of law reform: Mentally incapacitated adults*, published in 1995 (Law Commission, 1995). The draft Bill attached to that report did not make reference to sex, save to maintain the policy position^9^ that no person should be able to consent on behalf of another to sexual relations. We will pick up below how this position was translated into law in the shape of s.27 Mental Capacity 2005, but to continue the story chronologically, we need to stay with the 1990s.

In 1995, the Law Commission not only published its report on mental incapacity, but also a consultation paper on *Consent in the Criminal Law* (Law Commission, 1995). When, in 1999, the Home Office started a review of sexual offences, the Law Commission was asked to draw upon that work with a specific focus upon consent in sexual offences. The model of sexual capacity that the Law Commission initially proposed in response to the request was based very closely on the recommendations that it had developed in 1995 in its Mental Incapacity Report. However, in its final Report on Consent in Sex Offences (‘the Law Commission Report’), the Law Commission took a different approach (Law Commission, 2000). In Part IV, containing a detailed discussion of the issues arising in the context of mental incapacity, it recognised – after consultation – that:

*We do not believe that it is possible simply to transfer the existing tests, recommended for the assessment of mental incapacity for the purpose of **substitute** decision-making in the **civil** law, without change, into the criminal law. First, the civil law is not administered, so far as the fact-finding process is concerned, largely by lay people – juries and lay magistrates, for whose benefit the relevant tests need to be made clear and simple. Second, in the civil law, some of the issues which a person requires the capacity to understand may be very complicated, and we were anxious to protect those who might appear to understand but could not retain the necessary information for long enough to make a proper decision. Third, the obligations under the European Convention on Human Rights associated with sexual autonomy have an impact on our recommendation.*

(Law Commission, 2000, para 4.58) (footnotes omitted)

The Law Commission identified that the structure and language of the definition that it had proposed in the civil sphere did not easily translate into the criminal sphere and could be simplified to reflect “*the process of deciding to consent to sexual activity, as opposed to deciding upon a course of action with civil legal consequences. Essentially, this is because it is perceived to be a visceral, rather than a cerebral, process of decision-making*” (Law Commission, 2000, para 4.59). The Law Commission then identified that there were specific reasons why retention of information (relevant to its proposed civil test^10^) should not be included in any criminal test. As the Law Commission noted at paragraph 4.60:

[t]*he criminal law is concerned with whether or not a valid consent has been given. If A, who is unable to retain much information, decides to consent, on the basis of so much of the relevant information as he or she is capable of remembering, why should A be deemed incapable to make that decision if, when making it, he or she understood (for example) with whom sexual intercourse would take place, and wanted this to occur? In the substitute decision-making structure designed for the civil law, there are good reasons why, in the interests of that person’s financial, property, domestic and other affairs, he or she should have the benefit of a substitute decision-maker’s assistance. In the criminal law, however, rather than being of benefit to A, by providing a substitute decision-maker, the effect of such a requirement may be harmful to A, by denying him or her the autonomy to give consent to something about which he or she may properly be recognised as having sufficient information and understanding to make a choice* [in a footnote to the end of this last sentence, the Law Commission added “*Eg to have sexual contact with A’s husband, although their mental state is such that they are, at times, unable to remember much*.]^11^

A detailed discussion then followed of the potential problems caused by the requirement to understand reasonably foreseeable consequences, in particular in the case of a person unable to understand the consequences of pregnancy flowing from sexual intercourse. The Law Commission then proposed a statutory test for determining capacity for purposes of any non-consensual sexual offence, i.e., “*that a person should be regarded as being unable to make a decision on whether to consent to an act if (a) he or she is unable to understand (i) the nature and reasonably foreseeable consequences of the act, and (ii) the implications of the act and its reasonably foreseeable consequences; or (b) being able so to understand, he or she is nonetheless unable to make such a decision*.” For these purposes, “‘*mental disability’ should mean a disability or disorder of the mind or brain, whether permanent or temporary, which results in an impairment or disturbance of mental functioning*.” (Law Commission, 2000, para 4.84).

Further, the Law Commission returned to the dilemma of how to secure the autonomy of those with “mental disabilities” to engage in sexual activity in non-exploitative relationships, identified in its 1991 work, but not further addressed in its work leading towards the draft Mental Incapacity Bill. The Law Commission identified that:

*4.69 Sexual autonomy includes a right to refuse unwanted sexual attention (a negative aspect of this concept) as well as the right to choose to engage in sexual activity (a positive aspect). The difficulty is that a person’s mental disability may render them unable to refuse that attention as effectively as those without such disability. In cases such as those with severe learning disabilities, this risk is likely to continue throughout their lifetime. This vulnerability may be a result of:*

(a) *an inability to remove themselves from the risk;*
(b) *an inability to conceptualise or verbalise the abuse;*
(c) *lack of sex education, without which they may not have sufficient knowledge or understanding about sex and sexual relationships to make an informed choice; or*
(d) *low self-esteem, which results in a lack of belief that they have the right to refuse sex or a particular sexual partner.*

*4.70 If this negative aspect of sexual autonomy is to have any real meaning for those to whom these factors are material, the criminal law needs to provide protection for them. The positive aspect of sexual autonomy (freedom to engage in sexual activity) may be met by specific provision in the substantive criminal law* […].

The Law Commission proposed that there should be “*provision in the substantive criminal law of exemptions which recognise that, in certain circumstances, no offence would be committed despite a lack of capacity to consent. Such exemption might relate to apparently consensual sexual activity that takes place between two people, each with want of capacity, in non-exploitative circumstances; or where only one party to such activity lacks capacity, and this occurs in non-exploitative circumstances*” (Law Commission, 2000, para 4.72).

Recognising that it was venturing into difficult territory here, the Law Commission observed that, “*ultimately the question of where the appropriate balance should be struck, between the need to provide mentally disabled people with protection from abuse and the need to give recognition to their right to sexual or physical expression, must be for Parliament to decide, after wide consultation with those concerned with the mentally disabled*” (Law Commission, 2000, para 4.73). The Law Commission also identified that “*should Parliament choose to recognise that lawful sexual activity may take place between a person who lacks capacity to consent due to mental disability and one who does not, a limited exemption to criminal liability will be required*” (Law Commission, 2000, para 4.77); the Law Commission recognised the potential for this to give rise to an ‘abuser’s charter,’ such that it proposed that the law as it then stood be reformed to permit a defence where the defendant was able to establish that the victim was willing and there were no circumstances of oppression (Law Commission, 2000, para 4.78).

The Law Commission’s work fed into the (independent) Home Office Review of sex offence, *Setting the Boundaries: Reforming the law on sex offences* (Home Office, 2000) (‘the Review’). The Review recommended accepting the Law Commission’s proposal as to how to identify whether a person had or lacked capacity for purposes of non-consensual sexual offences (Recommendation 30). Under the sub-heading ‘*Sexual abuse by other people with a mental disability*,” the Review identified that:

*4.7.2 This is a very difficult area to address in law because of the complexity of legal processes when both the victim and the perpetrator are vulnerable adults. Such acts should not be considered outside the law, but if the perpetrator is severely handicapped he may not have the capacity to know that what he was doing was wrong, or indeed that his actions were unwanted. In such a case there would be no criminal intent. On the other hand it seems wrong for the criminal law to withdraw completely from this area. **If an abuser is capable of telling right from wrong, and has the capacity to know about sex and understand broadly its consequences, he or she should not be immune from prosecution, nor should such behaviour be condoned by carers**. We thought that providing definitions of capacity would help in deciding whether or not prosecution would be justified. It should be for the judgement of experts assisting investigators and prosecutors to determine whether there was sufficient lack of capacity by a perpetrator to justify a lack of intent.* [emphasis added]

The Government published a White Paper responding to the Review, *Protecting the Public: Strengthening protection against sex offenders and reforming the law on sexual offences* (Home Office, 2002). The approach set out in Chapter 4, dealing with special protection for children and the vulnerable, did not specifically address the proposals put forward by the Law Commission for determining whether a person had or lacked capacity for purposes of non-consensual sexual offences. Nor did it pick up the observations in the Review about the utility of capacity in the context of sexual abuse perpetrated by a person with a cognitive impairment. The White Paper took a different approach to the question of the criminalisation of sexual activity with individuals with “mental disabilities,” proposing an approach which moved from a situation where sexual activity with a person with a particular condition was automatically unlawful, to a situation where the sexual activity would be unlawful if the person, as a result of mental disorder, lacked capacity to choose to agree to the act or to communicate their choice.

Completing the legislative journey started by the Home Office review, the SOA 2003 received Royal Assent on 20 November 2003 and came into force on 1 May 2004. It re-defined some offences and created a range of new offences and made express provision in relation to the key issue of consent. Further, it established a range of new measures intended to guard against the commission of further offences by sex offenders and civil orders against an individual who has not been convicted of an offence but who nevertheless is thought to pose a risk of harm.

The SOA 2003 is divided into three parts; Part 1 (ss.1-79) makes a series of new provisions about sexual offences, Part 2 contains measures for protecting the public from sexual harm, and Part 3 contains general provisions relating to the Act, including minor and consequential amendments and commencement provisions.

As enacted, the SOA 2003 contains a general provision – s.74 – in relation to consent, providing that for purposes of Part 1 (providing for sexual offences), “a person consents if he agrees by choice, and has the freedom and capacity to make that choice.” This is the first statutory definition of consent in the context of the law relating to sexual offences. However, and despite the recommendation of the Law Commission and the Home Office Review, the SOA 2003 did not further define “capacity” in this context.

The SOA 2003 **did** define capacity in the context of the offences created in ss.30-33 in relation to persons with a mental disorder impeding choice. Each reference in ss.30-33 is in materially identical terms, providing that the person is to be taken to be unable to refuse if “*he lacks the capacity to choose whether to* [do the material thing] *whether because he lacks sufficient understanding of the nature or reasonably foreseeable consequences of what is being done, or for any other reason)*.”

How these provisions have been interpreted by the courts is dealt with in section four, below. Before we get there, however, and to remain true to the chronology, we need to note the parallel statutory developments in the shape of the passage of the MCA 2005.

## 3 The MCA 2005

The MCA 2005 created a statutory scheme for responding to the situation of a person over 16 who had impaired decision-making capacity. Importantly, though, and perhaps a fact not always sufficiently appreciated, it is a rather more limited Act than its name suggests. In particular, it is not an act setting down the definition of mental capacity for all purposes in English law. The statutory test of mental capacity it established in ss.2-3 expressly states that it is to apply “for purposes of this Act.” Those purposes are, primarily, enabling a ‘work-around’ – in the form of the defence contained in s.5 MCA 2005 – for the situation where a person appears to be unable to give the consent required to render a step taken in relation to their care and treatment lawful.^12^ Many other areas of the law retain their own tests for deciding whether the person has the requisite mental capacity to take a step with legal effect, examples including making a will (*Banks* [1870]),^13^ entering into a contract (*Boughton* [1873]), or making a gift (*Re Beaney* [1978]).

The MCA 2005 did not set down a statutory test in relation to incapacity in the sexual context. Nor was the SOA 2003 amended to make reference to the test in the MCA 2005, in circumstances where significant numbers of other legislative instruments were amended to make such reference.^14^ Indeed, with one exception, the MCA 2005 has nothing to say about sex. That exception relates to the legislative enactment of the policy line identified by the Law Commission in its work in the 1990s that certain categories of decision were so personal to the individual that no-one should be able to make them on their behalf. The Lord Chancellor’s Department consultation paper “*Who Decides? Making Decisions on behalf of Mentally Incapacitated Adults*” (1997) noted but did not expressly address the Law Commission recommendations (Lord Chancellor’s Department, 1997, para’s 7.17-7.20). The Government’s conclusions following that consultation “*Making Decisions: The Government’s proposals for making decisions on behalf of mentally incapacitated adults*” (Lord Chancellor’s Department, 1999), provided that “*there will be a number of decisions which no one may take on behalf of the person without capacity, including when acting under a general authority*,” including “*consent to sexual relations*” (Lord Chancellor’s Department, 1999, para 1.23). This then tracked though into the Draft Mental Incapacity Bill put to Parliament for pre-legislative scrutiny in 2002, clause 26 providing that “[n]*othing in this Act permits a decision on any of the following matters to be made on behalf of a person— (a) consent to marriage; (b) consent to have sexual relations* […].” Clause 26 was not expressly considered by the Joint Committee convened to conduct pre-legislative scrutiny on the draft Bill, and the provisions of clause 26 then made their way (in slightly modified form) into s.27, which provides in material part as follows:

(1) *Nothing in this Act permits a decision on any of the following matters to be made on behalf of a person—*
  (a) *consenting to marriage or a civil partnership,*
  (b) *consenting to have sexual relations,*[…]

Most probably because the question of the position of a person who wished to initiate sexual activity had never come into sharp legal focus before, there had prior to 2021 been very little jurisprudence about precisely what “consent” means for purposes of s.27 MCA 2005. At appellate level, the Court of Appeal in *Re M (An Adult) (Capacity: Consent to Sexual Relations)* [2015] Fam 61 considered that s.27 made plain that “*where a court finds that a person lacks capacity to consent to sexual relations, then the court does not have any jurisdiction to give consents on that person’s behalf to any specific sexual encounter* (*Re M*, para 78); an observation endorsed by Lord Stephens in *JB*. In similar vein, and at first instance, Hayden J observed in *London Borough of Tower Hamlets v NB* [2019] COPLR 398 at paragraph 34 that “[t]*he unambiguous effect of* [s.27] *is to strip the Court of any power to sanction a sexual relationship between P and another individual, in all circumstances, where it is established that capacity to consent to a sexual relationship does not exist. This is self-evidently necessary for a variety of ethical, moral and legal reasons*.” In *Re AA (Court of Protection: Capacity to Consent to Sexual Practices)* [2021] COPLR 14, Keehan J was required to consider the question of whether a young man had capacity to make decisions regarding auto-erotic asphyxiation (AEA). This appears to have been treated separately to the question of whether he had capacity to consent to sexual relations (*Re AA*, para 33). Keehan J concluded that there was reason to believe that the man did not have capacity to make decisions in respect of engaging in AEA, but accepted *“the agreed position of the parties that no best interest decisions fall to be made because it would be contrary to s.27(1)(b) or, at least, the philosophy of this provision for the court to make a decision in respect of AEA on AA’s behalf*.” (*Re AA*, para 55). In other words, Keehan J appears to have accepted that the “philosophy” of s.27(1)(b) should apply not solely to questions of giving consent on behalf of a person to sexual relations with another, but more broadly to decisions in relation to how an individual could or should obtain sexual release.

## 4 The defendant’s cognitive impairments

As both the Law Commission in the 1990s and the Home Office review of sexual offences had identified, it is quite possible for a person with cognitive impairments to be a (potential) perpetrator of as well as a victim of unwanted sexual activity. The Review had used the concept of capacity in this context (Home Office, 2002, section 4.7). The SOA 2003 makes no express reference to this term, nor does it adopt the proposal of the Law Commission for decriminalising “non-consensual, but non-exploitative” sexual relations involving one or the other party lacking the relevant decision-making capacity.

In the context of the carrying out of sexual acts to which the complainant could not or did not consent, the individual’s cognitive impairments will, in practice, be of relevance in one of six ways.

### First

in determining whether a prosecution should be brought, in particular through the application of the public interest stage of the ‘full code’ test set out in the Code for Crown Prosecutors (Crown Prosecution Service, 2018) which applies where the prosecutor has made an objective assessment that there is a realistic prospect of conviction (Crown Prosecution Service, 2018,, para 4.5).^15^ The Court of Appeal in *R v A(G)* [2014] 1 WLR 2469 expressed – obiter – concerns as to whether the public interest had been served in charging the defendant in question, who had a mild learning disability, with rape and then sexual assault on the particular facts of the case, describing the decision as ‘astonishing,’ a description conceded with hindsight by prosecuting Counsel (*R v A(G),* para 15).

### Second

in determining whether the defendant could be said to have “reasonably believed”^16^ that the complainant consented for purposes of the non-consensual sexual offences created by the SOA 2003 (and the evidential presumption about consent set down in s.75 2003). As a general proposition, and as held by the Court of Appeal in *R v B (MA)* [2013] 1 Cr. App. R. 36, unless the defendant’s state of mind amounted to insanity in law “*beliefs in consent arising from conditions such as delusional psychotic illness or personality disorders must be judged by objective standards of reasonableness and not by taking into account a mental disorder which induced a belief which could not reasonably arise without it* (*R v B (MA),* para 40). However, the Court of Appeal continued:

*41. It does not follow that there will not be cases in which the personality or abilities of the defendant may be relevant to whether his positive belief in consent was reasonable. It may be that cases could arise in which the reasonableness of such belief depends on the reading by the defendant of subtle social signals, and in which his impaired ability to do so is relevant to the reasonableness of his belief. We do not attempt exhaustively to foresee the circumstances which might arise in which a belief might be held which is not in any sense irrational, even though most people would not have held it. Whether (for example) a particular defendant of less than ordinary intelligence or with demonstrated inability to recognise behavioural cues might be such a case, or whether his belief ought properly to be characterised as unreasonable, must await a decision on specific facts. It is possible, we think, that beliefs generated by such factors may not properly be described as irrational and might be judged by a jury not to be unreasonable on their particular facts. But once a belief could be judged reasonable only by a process which labelled a plainly irrational belief as reasonable, it is clear that it cannot be open to the jury so to determine without stepping outside the Act*

(*R v B (MA),* para 41).

As the editors of *Rook & Ward* (Rook & Ward, 2021) identify at paragraph 1.390: “[t]*his has left open the possible relevance of conditions such as autism spectrum disorder and Asperger’s syndrome, which might lead to an impaired or distorted perception of a complainant’s behaviour*.” They continue:

*There is a mounting body of evidence that a person with Asperger’s may misinterpret a victim’s response to sexual overtures.* […^17^] *It remains to be seen whether the courts will consider such a characteristic as relevant where there is evidence that because of Asperger’s disorder, a defendant has failed to appreciate cues and communications which would have alerted others that their behaviour was distressing or not resulting in consent. We suggest that, in contrast to an irrational belief arising from a psychotic belief which could never be reasonable, a misinterpretation of behaviour due to a development disorder such as Asperger’s falls into a different category. Such a condition is constant, causes significant impairment in social functioning, and can properly be taken into account by a jury.*

As to the dividing-line identified by the Court of Appeal at paragraph 41, the editors of *Rook & Ward* suggest (Rook & Ward, 2021, para 1.395) that:

*Characteristics such as a condition in which defendant has psychotic episodes and/or periods of delusional thinking will not be relevant. Nor will character defects such as alcoholism or excessive vanity. We suggest extreme youth at the time, and constant conditions such as blindness and learning disability, may be relevant to the extent that they have a bearing on a particular defendant’s ability to appreciate the risk that a person is not consenting to sex. It will then be a matter for the jury to decide whether any such characteristic has a bearing on the issue of reasonable belief.*

### Third

for purposes of deciding whether the person is unfit to plead, i.e., whether they have a disability which would constitute a bar to them being tried,^18^ at that point, by virtue of the operation of s.4A Criminal Procedure (Insanity) Act 1964, a jury will be empanelled in order to make judgment as to whether or not the defendant had committed the act alleged. *R v A(G)* [2014] 1 WLR 2469 was precisely such a case, which meant that the jury in the case was not required to determine the mens rea of the defendant and not required to consider whether he did, or had reason to, believe that the complainant was consenting to his sexual advances (*R v A(G),*para 13). In such a case, the court will have the powers of disposal available under s.5 Criminal Procedure (Insanity) Act 1964, i.e. ‘diversion’ into the psychiatric system under the Mental Health Act 1983 or an absolute discharge.

### Fourth

determining whether the individual should be made the subject of a special verdict that they are not guilty by reason of insanity. The Court of Appeal in *R v B (MA)* [2013] 1 Cr. App. R. 36 left open the potential for the insanity defence to apply in the context of non-consensual sexual situations, although there is no reported case where it has been applied. Again, in such a case, the court will have the powers of disposal available under s.5 Criminal Procedure (Insanity) Act 1964, i.e., ‘diversion’ into the psychiatric system under the Mental Health Act 1983 or an absolute discharge.

It should be noted in relation to the third and fourth of these matters that the Law Commission has proposed reforms to both aspects (see Law Commission 2013 and Law Commission 2015), drawing upon the concept of capacity, but in both proposing re-purposing the concept as found in the MCA 2005 to meet the policy goals of the criminal law.^19^ In criticising these proposals, Alec Buchanan has helpfully highlighted one of the key distinctions between the different functions of capacity in civil and criminal contexts:

*In* […] *non-criminal cases the context is one where 1) a choice remains to be made or acted upon, 2) the consequences of that choice are not meant to influence the court’s decision and 3) the important question for the court is who shall be allowed to make the choice. In criminal cases where a mental state defence is being offered 1) the choice in question has already been made and acted upon, 2) there is no “value neutrality” because the law has already declared the act illegal and 3) the question is whether the defendant should be punished in the usual way. A concept that has contributed to the civil law of decision-making is not automatically useful in criminal law.*

*References to a defendant having mental capacity should be included in a replacement for the insanity defence if they help us identify non-responsible defendants. I do not think that the references to capacity in the Law Commission’s proposals would prevent courts from arriving at the right decisions, even in difficult cases. Rather I see the proposed wording as a missed opportunity to express the criteria for permitting a legal excuse in the simplest language possible. The Law Commission document introduces a complicated abstraction, capacity, that has been defined in several different ways in the literature on the jurisprudence of excuse and that if challenged in court will have to be defined again*.

(Buchanan, 2015).

### Fifth

if a defendant with a cognitive impairment is convicted of an offence, and not diverted into the psychiatric system, their impairments would be relevant in determining his sentence: the Sentencing Council Guidelines include as a mitigating factor “Mental disorder or learning disability,” particularly where this is linked to the commission of the offence (Sentencing Council, 2014, Step 2).

### Sixth

and (in one sense) separately, the fact that a person is considered to have cognitive impairments will be relevant as to whether either Sexual Risk Orders (‘SROs’) and Sexual Harm Prevention Orders (‘SHPOs’) under the SOA 2003 are available. The former can be applied for under s.103A SOA 2003 where the person has committed an offence (even if they are found to be unfit to plead or because they can rely upon the insanity defence). An SRO can only be applied where the person has done an act of a sexual nature (s.122A(2)). However, where the person cannot understand a term or terms contained within either an SRO or an SHPO, such should not be sought from the criminal courts, because that person would not pass the test of necessity for public protection.^20^

## 5 Sexual capacity before the criminal courts: 2003-2021

Prior to 2003, the common law had *“developed no clear principles governing whether a person had capacity to consent to sexual acts*” (Rook & Ward, 2021, para 1.226). In 2006, in *X City Council v MB, NB and MAB* [2006] 2 FLR 968 (para 82), Munby J gave his (obiter) observations as to the meaning of capacity to consent at common law, namely*: “the capacity to choose, to decide whether to give or withhold consent, dependent upon the capacity to understand the nature and character of the act, crucially, the capacity to understand the sexual nature of the act.”*

In *R v Cooper (Gary Anthony)* [2009]1 WLR 1786, concerned with s.30 SOA 2003, Baroness Hale – who had led the Law Commission’s Mental Incapacity work in the 1990s – addressed the different approaches of the MCA 2005 and the SOA 2003; see paragraphs 29-30, where, discussing s.30 SOA 2003 (mirrored in ss.31-3), she identified that it embodies a “significant difference” to that contained in ss.2-3 MCA 2005.^21^ She also said she was “*far from persuaded*” that Munby J’s observations in *Re MAB* and *Re MM* as to the common law test were correct (*R v Cooper* [2009], para 24). However, ultimately, she did not have to resolve these doubts as she considered that the statutory test contained in s.30 SOA 2003 had put matters beyond doubt.

The question of capacity for purposes of the SOA 2003 arose again in *R v A(G)* [2014] 1 WLR 2469. This case concerned a defendant with a moderate learning disability who had been convicted of sexually assaulting a complainant with a mild learning disability, contrary to s.30 SOA 2005. A prosecution expert had concluded that the complainant lacked capacity to consent to sexual relations pursuant to ss.2-3 MCA 2005. The defendant was found to be unfit to plead to the offence,^22^ but a jury found that he had committed the alleged assault.^23^ The defendant’s appeal was successful. The Court of Appeal *R v A(G)* [2014] 1 WLR 2469 interpreted the term ‘capacity’ in s.74 by reference to ss.30-33 (*R v AG*, para’s 24-25). Macur LJ stated:

*24 The Sexual Offences Act 2003 does not explicitly define capacity nor refer to a definition of the same within the meaning of this* [Mental Capacity] *Act. Section 74 of the 2003 Act provides the interpretation of “consent”: “For the purposes of this Part, a person consents if he agrees by choice, and has the freedom and capacity to make that choice”.   Quite clearly, therefore, the issue of consent involves inter alia the capacity to choose, subject to inferences that may be drawn in accordance with sections 75 and 76 of the Act. Sections 30(2)(a), 31(2)(a), 32(2)(a) and 33(2)(a) of the 2003 Act which create offences against persons with mental disorder impeding choice express a lack of capacity as follows:*

*“he lacks the capacity to choose . . . (whether because he lacks sufficient understanding of the nature or reasonably foreseeable consequences of what is being done, or for any other reason) . . .”*

*25 The bracketed words reflect the provisions of sections 2(1) and 3(1) of the 2005 Act, and lead us to determine that the difference in definition of capacity in the civil and criminal jurisprudence is a difference without distinction.*[^24^]

*26 However, the similarity of definition does not, in our view, dictate the same standard of proof. We observe that the adjudications of the Court of Protection will look to the future in generality; the criminal law looks retrospectively to specific acts of the past.*

We will return to the decision in *R v A (G)* below, but at this stage note that the Court of Appeal appear to made these observations without having been addressed upon any of the matters set out above.^25^

## 6 Sexual capacity before the civil courts

A comprehensive review of the case-law before the case of *JB* falls outside the scope of this paper; it was conducted by Baker LJ in the Court of Appeal decision in *JB* ([2020] EWCA Civ 735, [2021] Fam 37) between paragraphs 24 and 75. Two overarching themes appear from those cases.^26^ The first is a protracted judicial disagreement about whether the relevant test was a so-called ‘act’ (or ‘issue’) specific or person-specific, resolved (prior to *JB*) by the Court of Appeal in *In re M (An Adult) (Capacity: Consent to Sexual Relations)* [2014] EWCA Civ 37, [2015] Fam 61 in favour of an act-specific test. The second is a tension between seeking to set a low bar to the test to promote autonomy and securing against the risk of exploitation, as to which see, most obviously, cases such as *Re TZ* [2013] EWCOP 2322, [2013] COPLR 477 and *Re TZ (No. 2)* [2014] EWCOP 973, [2014] EWCOP 973, [2014] COPLR 159. The (perhaps crude) fudge adopted in *Re TZ (No. 2)*, adopted subsequently in other cases, was to conclude that the person in question had capacity to consent to sexual relations, but not to make decisions about contact with others. Such a conclusion opened the way to regulating contact through the prism of best interests, and hence (in effect) to enabling only ‘safe’ sexual encounters to take place.

## 7 JB – the Supreme Court pronounces

The definitional disharmonies illustrated above were finally brought to the fore by the case of JB. At the centre of this case was a 38 year-old-man, JB, who had several physical and cognitive impairments, and in whose life the local authority had significant involvement. The local authority’s concern in this case was that JB would be at risk of engaging in a course of sexual activity which may not have the consent of any prospective sexual partner, in circumstances where the evidence was that his “number one priority” was “to get” a woman as a sexual partner.^27^ The evidence was also that JB had sought sexual activity with women who, potentially, either would not, or could not, consent.

The local authority therefore brought proceedings before the Court of Protection for declarations and decisions which would enable it to exercise control over JB’s contact so as to prevent him having unsupervised contact with any woman. At first instance, the local authority and the Official Solicitor as JB’s litigation friend reached agreement on the majority of issues relating to JB’s capacity, save for the question of whether he had capacity to consent to sexual relations. Roberts J held^28^ that he did have such capacity; the local authority appealed to the Court of Appeal which held^29^ that (1) the proper question was whether he had capacity to decide to engage in sexual relations; and (2) he lacked such capacity. The Official Solicitor – acting as JB’s litigation friend^30^ – appealed on JB’s behalf to the Supreme Court.

Before the Supreme Court, the critical question was whether, in order to have capacity to engage in sexual relations, JB needed to understand that the other person must be able to consent, and gives and maintains consent throughout, to the sexual encounter in question. Expert evidence was adduced to indicate that, by virtue of his Autistic Spectrum Disorder, JB could not understand, use, or weigh this information. The question for the Supreme Court was whether this information was legally relevant to a decision to engage in sexual relations for purposes of the capacity test contained in s.2 MCA 2005. If the information was legally relevant to the decision, then (assuming that, upon further examination, it remained the case that JB could not understand, use, or weigh it), JB would not have the requisite mental capacity to make the decision to engage in sexual relations. He would thus – to quote Lord Stephens (who gave the Supreme Court’s sole judgment) – be ‘deprived of all sexual relations’ as no other person would be entitled to make this decision on his behalf by virtue of s.27 MCA 2005.^31^

The Supreme Court held, unanimously, that information about the other person’s consent was legally relevant to the question of whether JB had capacity to decide to engage in sexual relations. However, as with the Court of Appeal, it declined to make a final declaration that JB lacked this capacity, but rather remitted the matter to Roberts J for her to consider the test, and any further expert evidence required to address the test, in light of its judgment.^32^

The focus of the Supreme Court’s decision in *JB* was upon the requirements of the capacity test under the MCA 2005. However, the criminal position was squarely before it.^33^A central limb of the Official Solicitor’s challenge to the Court of Appeal’s decision was that it had created a test with an “impermissible difference between the civil and criminal law” ( *JB*, para 97). Lord Stephens rejected this challenge. He identified that there were differences in the application of the test for capacity which may lead to different conclusions. The first was the different standards of proof (*JB*, para 100). The second was the fact that:

*the focus of the criminal law, in the context of sexual offences, is retrospective. It focuses upon a specific past event. Any issue relating to consent is evaluated in retrospect with respect to that singular event. So, the material time in a criminal case is the time of the alleged offence and the question becomes, for instance, “Did P have capacity to consent at that time?” But a court assessing capacity to engage in sexual relations under the MCA ordinarily needs to make a general, prospective evaluation which is not tied down to a particular time*

(*JB*, para 101).^34^

Lord Stephens “*agree*[d] *that all else being equal, it is in principle desirable, though not necessary, that there should be the same test for capacity in both the civil and criminal law*” (*JB*,para 103), and there were “sound policy reasons” why the civil and criminal law test for capacity should be the same. He considered that Munby J had set out the reasons of policy in *In re MM* (at para 89):

*In this context both the criminal law and the civil law serve the same important function: to protect the vulnerable from abuse and exploitation … Viewed from this perspective, X either has capacity to consent to sexual intercourse or she does not. It cannot depend upon the forensic context in which the question arises, for otherwise, it might be thought, the law would be brought into disrepute.*

Lord Stephens agreed, finally, that the civil law test for consent could not impose a less demanding test of capacity than the criminal law test (*JB*, para 105). However, he considered that it remained possible for the civil law to impose a more demanding test, based upon the “countervailing and overriding policy reasons” of protecting others and the person themselves (*JB*, para 106). Whilst he agreed with Munby J that in general terms, the criminal and civil law served the same function, he also considered that that should not concern the different purposes of the two branches of the law and the ways in which they carried out their functions, such that “it may be permissible to adopt different tests of capacity in the civil and the criminal law” (*JB*, para 107).

Importantly, however, Lord Stephens made clear that whether the clarification of the test of capacity under the MCA 2005 in *JB* resulted in any differences with the test for capacity in the criminal law “is best left to be decided on the facts of individual criminal cases and may turn on the particular criminal offence in question” (*JB*, para 108).

That self-direction did not prevent Lord Stephens making a number of obiter observations about whether there were any differences between civil and criminal law.

### First

at paragraphs 110-1, Lord Stephens disagreed with Macur LJ in *R v A (G)* that the difference in definition of capacity in the civil and criminal jurisprudence is a difference without distinction:

*For instance, in the context of the MCA, information relevant to the decision to “engage in” sexual relations includes the fact that the other person must have the ability to consent to the sexual activity and must in fact consent before and throughout the sexual activity. P lacks capacity if P is unable to understand that information or if he is unable to use or weigh it as part of the decision-making process. However, to my mind that aspect of capacity is irrelevant in the context of sections 30-33 SOA provided P is the complainant rather than the alleged perpetrator* […]*. In that criminal context P’s capacity to consent would not include a sufficient understanding by him of the fact that the alleged perpetrator (that is, the other person) must have the ability to consent to the sexual activity and must in fact consent before and throughout the sexual activity.*

### Second

in relation to the position of P as complainant in respect of offences outside ss.30-3 SOA 2003 (for instance, rape), Lord Stephens considered that the primary issue would relate to P’s capacity to consent to, not engage in sexual relations. These were, he considered (*JB*, para 112), two different concepts:

*The capacity to “engage in” sexual relations encompasses both P as the initiator of those relations and P as the person consenting to sexual relations initiated by another. The information relevant to a decision whether to initiate sexual relations includes the fact that the other person must have the ability to consent to the sexual activity and must in fact consent before and throughout the sexual activity. That is not information relevant to an evaluation of whether P has the capacity to “consent to” sexual relations initiated by another person. As the Court of Appeal stated in this case (at para 93) “The word "consent" implies agreeing to sexual relations proposed by someone else.” The capacity to consent to sexual relations for the purposes of the criminal law is concerned with the understanding of the complainant (whom I have been referring to as P) about matters which are relevant to their autonomy, not those which are relevant to the autonomy of the alleged perpetrator. I do not consider that the criminal law requires that a complainant understands that their assailant must have the capacity to consent and in fact consents before the complainant can be considered to have capacity. I do not discern any difference in this regard between the civil and criminal law.*

### Third

Lord Stephens rejected the Official Solicitor’s argument that the Court of Appeal’s approach (in the civil context) required more than was expected of individuals in the criminal law context when it comes to determining whether they had a reasonable belief in the consent of the other (*JB*, para’s 113-4). Lord Stephens noted the decision of the Court of Appeal in *R v B (MA*) [2013] as to the potential relevance of a person’s inability to read subtle social signals, but made clear that he was not intending either to contradict or build upon that judgment (*JB*, para 116).

### Fourth

Lord Stephens identified the position where the individual with cognitive impairments might be accused of an offence under ss.30-33 SOA 2003. He observed that that the concept of engaging in sexual activity (central to each of these offences) “is a descriptor of the actus reus of that offence and is consistent with the clarification of the law in respect of the MCA which also uses the concept of engaging in sexual relations. I do not discern any difference in that respect between the civil and criminal law” (*JB*, para 116). He considered that, for purposes of the mens rea, the accused’s “knowledge of the complainant being unable to refuse includes the reasonably foreseeable consequences of what is being done but it does not include a requirement that the complainant should have any understanding of the fact that the alleged perpetrator (that is, the other person) must have the ability to consent to the sexual activity and must in fact consent before and throughout the sexual activity” (*JB*, para 116). Again, therefore, he could not discern any difference between the civil and criminal law.

## 8 Conclusion: where are we now, and where might we go?

As a starting point, we should make clear that we respectfully agree with Lord Stephens’ conclusion that there is no necessary requirement that the tests of capacity should be the same for purposes of the civil and the criminal law. Even if, in broad terms, the two concepts should march in track, this paper has traced how the SOA 2003 was developed along a parallel trajectory to the MCA 2005, and in circumstances where all the way through that process, it has been identified why there are sound reasons why they do not need to be slavishly aligned. It is, we suggest, unfortunate that the courts prior to the Supreme Court in *JB* were led to overlook how those policy reasons tracked through into statutory wording which was, intentionally, different.

Luckily, however, the resetting of the position by the Supreme Court in *JB* allows stock to be taken by the criminal courts in England & Wales going forward. In so doing, we suggest that it has left the terrain open for reconsideration of the concept of ‘capacity’ within the SOA 2003 for the following reasons.

First, save for noting Munby J’s (obiter) observation in *MAB* [2006] about the common law test seemingly having been preserved (*JB*, para 109), Lord Stephens did not address the meaning of the term ‘capacity’ for purposes of s.74 SOA 2003. Nor did he address Baroness Hale’s observations about the correctness of *MAB* [2006] in *R v Cooper* [2009].

Second, whilst Lord Stephens made (obiter) observations that the term ‘capacity’ in relation to the specific offences in ss.30-3 SOA 2003 “reflected” the language of ss.2-3 MCA 2005, but he expressly recognised the difference in wording between the two sets of provisions (*JB* [2021], para 110).

Third, the decision of the Court of Appeal in *R v A (G)* [2014] is, strictly, limited narrowly to the question of the relevant standard of proof, so its observations about the meaning of ‘capacity’ for purposes of s.74 and s.30-3 SOA 2003 were obiter. They are therefore not binding upon future criminal courts considering either s.74 or ss.30-3 SOA 2003. More fundamentally, we also suggest that they were observations reached per incuriam, as the court had not been addressed on material matters, including both the entirety of the relevant legislative provisions (both the MCA 2005 and the SOA 2003) and the observations of Lady Hale in *Cooper* [2009] about the interaction between the two.^35^

If, therefore, it is time to start again, we would respectfully suggest that the Humpty Dumpty rule should therefore apply, and those appearing before the courts and the courts themselves should take a fresh run at what they mean when they say ‘capacity’ in the SOA 2003, rather than simply importing automatically the test contained in s.2-3 MCA 2005.

More radically, we suggest that the SOA 2003 might usefully be revisited to examine whether the term ‘capacity’ is actually necessary in either of the places it appears: it could, for instance, be possible to use the term ‘ability,’ and then (if required) to specify what the constituent components of that ability might be. We are realistic about the prospect of statutory reform in the near term, but that does not mean that it should not be kept on the radar, especially if (for instance) the Law Commission’s broader work on capacity in the criminal context (Law Commission, 2013 and 2015) starts to bear legislative fruit.

Even more radically, and raising our eyes above the purely domestic horizon, we note that it might be said that everything that we have set out above could be considered entirely irrelevant. Whatever components of (mental) capacity are actually to be found in the relevant provisions of the SOA 2003, a person who did not have those functional components at the relevant time is, by operation of those provisions, deemed to have been unable to give consent (or express choice) effective as a matter of law to prevent criminal acts being committed. In other words, the lack of (mental) capacity gives rise to a lack of (legal) capacity to consent/choose. The UN Committee on the Rights of Persons with Disabilities considers that any such linkage is contrary to Article 12 CRPD (Committee on the Rights of Persons with Disabilities, 2014). The stance of the Committee and the status of the General Comment in which it expressed this position are both contested (Essex Autonomy Project, 2016). More fundamentally, it is not entirely obvious how the Committee would seek to square the circle in relation to sexual capacity,^36^ but the stakes are high. We suspect that it will be quite some time – if ever – before any jurisdiction seeking to comply with the Committee’s interpretation of Article 12 takes the equivalent step in the field of sexual consent to that taken in relation to voting in England & Wales in 2006 of abolishing any linkage between cognitive impairment and legal capacity.^37^ But for jurisdictions seeking to address the consequences of cognitive impairment in the field of sexual capacity – which might one day include England & Wales – the CRPD undoubtedly provides a stimulus for a much more radical reframing of the terms of the debate.

## Supplementary Material

Appendix

## Footnotes

2 See, e.g., Sir James Munby in *An NHS Trust* [2020] at paragraph 56.

3 That is not to say that it does not have profound implications in the civil context, the subject of forthcoming work by the same authors [ref removed for anoymisation purposes].

4 For the interaction between the two concepts, see, in particular, Essex Autonomy Project (2016). Seeking compliance with the UNCRPD also gives rise to difficult issues involving the interaction between Articles 12 and 16 (the right to freedom from exploitation, violence and abuse), discussed also in the work referred to in the footnote above.

5 Although we note that the concept of “instinctual” capacity that featured in cases such as R v Ryan (1846) does appear – on one view – to come close to some of the approaches advocated within the context of those relying on the UN Convention on the Rights of Persons with Disabilities to seek to reduce the number of obstacles towards universal recognition of the (legal) capacity of those with impairments.

6 Sections 7, 14(4) and 15(3) Sexual Offences Act 1956, and s.128 Mental Health Act 1983. A ‘defective’ was defined in s. 45 Sexual Offences Act 1956 as a person suffering from a state of arrested or incomplete development of mind, which included severe impairment of intelligence and social functioning within the meaning of the Mental Health Act 1959. As Sandland identifies (Sandland, 2023), there was very little caselaw relating to these offences; probing why such might have been the case would be a fascinating exercise.

7 The footnote to this sentence read “[t*]hus, it is rape to have intercourse with a girl or woman who cannot understand the implications of what is taking place. R. v. Howard* [1966] 1 W.L.R. 13.”

8 The footnote to this sentence referred to an academic paper: M.J. Gunn, “Sexual Rights of the Mentally Handicapped”, in E. Alves (ed.), Issues in Criminological and Legal Psychology No. 10: Mental Handicap and the Law, (1987).

9 In draft clause 30. See also paragraph 4.29 of the report.

10 I.e. the test contained in what became ss.2-3 MCA 2005, containing the requirement for the person to be able to understand, retain, use and weigh relevant information (and communicate their decision).

11 Interesting, the Law Commission appeared, at least in this discussion, to be contemplating the possibility of another person being able to give consent on behalf of the individual. However, in their work upon the civil aspects of mental capacity, the Law Commission had excluded this possibility.

12 The legislative history of the Act, and its background, is summarised in (Ruck Keene et al, 2019)

13 See also, most recently, *Re Clitheroe* [2021].

14 Schedule 6 MCA 2005.

15 A separate – important – issue arises here in relation to the extent to which a **complainant** with cognitive impairments is likely (1) to have identified and/or raised the fact of a potential offence; or (2) to be taken as a credible and reliable witness by the police and CPS.

16 Section 1(1)(c) SOA 2003 requiring the prosecution to prove that the defendant did not reasonably believe that the other consents.

17 The footnote here refers to (Freckleton and List, 2009).

18 Section 4(1) Criminal Procedure (Insanity) Act 1964.

19 In the context of the insanity defence, the Law Commission has proposed the new defence of “*not criminally responsible by reason of recognised medical condition*,” where the accused “*wholly lacked a relevant criminal capacity at the time of the alleged offence because of a qualifying condition*,” the relevant capacities being “*to form a judgment rationally, to appreciate the wrongfulness of the act or omission, or to control his or her physical acts in relation to what he or she is charged with having done*” (paragraph 4.2). The draft legislation appended to the *Unfitness to Plead* report (Law Commission 2015), the draft Criminal Procedure (Lack of Capacity) Bill, identifies specific abilities that the defendant must have to participate effectively in a trial (in cl.3) and to plead guilty (in cl.5), and for both sets down a test for identifying that ability which is very similar to the functional test in s.3(1) MCA 2005, but contains no equivalent to the balance of s.3, the so-called ‘diagnostic’ element in s.2, or the principles contained in s.1(1)-(3) MCA 2005.

20 See the observations of the editors of *Rook & Ward on Sexual Offences* (Rook and Ward, 2021) to the same effect at paragraphs 36.27 and 36.28 in relation to SHPOs, the ‘necessity’ test applying equally to SROs (s.122A(9))

21 Not least, as she identified, that a person could lack capacity to consent for purposes of the MCA 2005 but not lack capacity for purposes of ss.30-33 SOA 2003 if they did not have a mental disorder.

22 Section 4 Criminal Procedure (Insanity) Act 1964

23 Section 4A Criminal Procedure (Insanity) Act 1964

24 This observation was, in fact, obiter, as the case turned upon the standard of proof.

25 It is perhaps telling that the editors of Rook & Ward (2021) do not proceed on the basis that the question of what it means to have capacity to consent for purposes of s.74 SOA 2003 has been settled by *R v A(G)*: see paragraph 1.237. As they also observe,“[w]*here a defendant has had penetrative sex with a person who is unable to refuse because of, or for a reason relating to mental disorder, an appropriately serious offence may be charged which obviates the need to charge rape. This ensures that the jury does not have to grapple with issue of whether the complainant had capacity to consent*” (paragraph 1.238, the same logic also applying to other offences involving non-consensual sexual touching).

26 Stimulating commentary on the case-law can be found, in particular, in Sandland (2013), Herring and Wall (2014), Clough (2014), and Series (2015).

27 The evidence of Dr Thrift, independent consultant clinical psychologist, as to what JB had relayed to her; set out in the judgment of the Supreme Court at paragraph 23.

28 *Re JB (Capacity: Consent To Sexual Relations And Contact With Others)* [2019] EWCOP 39.

29 *A Local Authority v JB* [2020] EWCA Civ 735.

30 I.e. on his behalf, JB lacking capacity to conduct the proceedings.

31 *JB*, para 1.

32 There is no further reported judgment at time of writing, so we do not know whether Roberts J made such a declaration. It is also important to emphasise that the local authority was continuing work to support JB’s decision-making abilities (see paragraph 119 of the judgment of the Supreme Court).

33 Including through the presence of specialist criminal counsel on behalf of both appellant and respondent.

34 The prospective evaluation causes its own difficulties, not examined here.

35 The Court of Appeal referred (*R v A(G)* para 17) to the fact that the decision in *IM* addressed *Cooper*, but did not independently address the latter decision.

36 An academic closely associated with the Committee has sought to do so, but on the basis that “[i]*t is, of course, important to ensure that all parties in any sexual activity are agreeing to that sexual activity. That agreement cannot exist without the parties having the decision-making skills necessary to **understand**, to some extent, what the nature of the sexual activity is*” (emphasis added): that appears, in reality, to be a functional mental capacity test (Arstein-Kerslake, 2015).

37 Section 73 Electoral Administration Act 2006 providing that “[a]ny rule of the common law which provides that a person is subject to a legal incapacity to vote by reason of his mental state is abolished.”
