# Supplementary material for: What place ‘capacity’ in the criminal law relating to sex post JB?
Source: Int J Law Psychiatry. Author manuscript; Available in PMC 2023 Aug 17. (PMC7614960; doi:10.1016/j.ijlp.2022.101843)
Supplement: Appendix [file EMS182030-supplement-Appendix.pdf]

Appendix

List of cases referred to

1. *R v. Ryan* (1846) II Cox C C 115
2. *Banks v Goodfellow* (1870) LR 5 QB 549
3. *Boughton v Knight* (1873) LR 3 PD 64
4. *R. v. Howard* [1966] 1 WLR 13
5. *Re Beaney (Deceased)* [1978] 2 All ER 595
6. *X City Council v MB, NB and MAB* [2006] EWHC 168 (Fam)
7. *Re MM (an adult)* [2007] EWHC 2003 (Fam)
8. *R v Cooper (Gary Anthony)* [2009] 1 WLR 1786
9. *R v B (MA)* [2013] 1 Cr App R 36
10. *Re TZ* [2013] EWCOP 2322
11. *In re M (An Adult) (Capacity: Consent to Sexual Relations)* [2014] EWCA Civ 37
12. *R v A(G)* [2014] 1 WLR 2469
13. *Re TZ (No. 2)* [2014] EWCOP 973
14. *London Borough of Tower Hamlets v NB* [2019] EWCOP 27
15. *Re JB (Capacity: Consent To Sexual Relations And Contact With Others)* [2019] EWCOP 39.
16. *An NHS Trust v X (No 2)* [2020] EWHC 65 (Fam)
17. *A Local Authority v JB* [2020] EWCA Civ 735
18. *A Local Authority v JB* [2021] UKSC 52
19. *Clitheroe v Bond* [2021] EWHC 1102 (Ch)
20. *Re AA (Court of Protection: Capacity to Consent to Sexual Practices)* [2021] COPLR 14
